# A protocol to evaluate RNA sequencing normalization methods

**DOI:** 10.1186/s12859-019-3247-x

**Published:** 2019-12-20

**Authors:** Zachary B. Abrams, Travis S. Johnson, Kun Huang, Philip R. O. Payne, Kevin Coombes

**Affiliations:** 1 Department Biomedical Informatics, Ohio State University, 250 Lincoln Tower, 1800 Cannon Dr. Columbus, Columbus, OH 43210 USA; 2 Department of Medicine, Indiana University School of Medicine, 545 Barnhill Drive, Indianapolis, IN 46202 USA; 3 Regenstrief Institute, Indiana University, 1101 West 10th Street, Indianapolis, IN 46262 USA; 4 Department of Biomedical Informatics, Washington University, 4444 Forest Park Ave, Suite 6318 Campus Box 8102, St. Louis, MO 63108-2212 USA

**Keywords:** RNASeq, Normalization, Standardization, Biological variability

## Abstract

**Background:**

RNA sequencing technologies have allowed researchers to gain a better understanding of how the transcriptome affects disease. However, sequencing technologies often unintentionally introduce experimental error into RNA sequencing data. To counteract this, normalization methods are standardly applied with the intent of reducing the non-biologically derived variability inherent in transcriptomic measurements. However, the comparative efficacy of the various normalization techniques has not been tested in a standardized manner. Here we propose tests that evaluate numerous normalization techniques and applied them to a large-scale standard data set. These tests comprise a protocol that allows researchers to measure the amount of non-biological variability which is present in any data set after normalization has been performed, a crucial step to assessing the biological validity of data following normalization.

**Results:**

In this study we present two tests to assess the validity of normalization methods applied to a large-scale data set collected for systematic evaluation purposes. We tested various RNASeq normalization procedures and concluded that transcripts per million (TPM) was the best performing normalization method based on its preservation of biological signal as compared to the other methods tested.

**Conclusion:**

Normalization is of vital importance to accurately interpret the results of genomic and transcriptomic experiments. More work, however, needs to be performed to optimize normalization methods for RNASeq data. The present effort helps pave the way for more systematic evaluations of normalization methods across different platforms. With our proposed schema researchers can evaluate their own or future normalization methods to further improve the field of RNASeq normalization.

## Background

Several RNA sequencing (RNASeq) technologies provide transcriptomic expression data, but the data may vary based on a variety of often-uncontrollable experimental conditions [1]. Consequently, RNASeq raw data needs to be adjusted so that comparisons are based on biological truth. This mathematical adjustment is known as normalization. Multiple normalization methods exist and method selection depends on 1) the type of genomic data, 2) the platform (e.g. Illumina, Life Sciences, or ArrayCGH) originally used to collect the data, 3) the scale of the data, and 4) the planned downstream analyses.

Previous studies have compared normalization methods to determine which method best preserves biological reality while reducing experimental noise [2]. Most of these experiments were conducted on small, heterogeneous data sets that were not collected for the specific purpose of systems level evaluation [3, 4]. Rather, they were conducted on publicly available, secondary data, which often lacked an a priori experimental structure, an adequate sample size, and/or sufficient technical replicates for meaningful results at multiple scales. The a priori experimental structure is especially important because very few datasets are designed for evaluation of sequencing technologies and algorithms. Critically, there is also a lack of standardized tests to evaluate the various normalization methods commonly employed.

The most common way to evaluate normalization techniques is to compare the results of raw and normalized data to quantitative real-time PCR (qPCR), which is considered the gold standard in terms of true expression values [2]. With respect to processed sequencing data, qPCR can be used as a ground truth. However, this is only one facet of how normalization should be evaluated because it is only looks at one aspect of a multifaceted statistical problem.

In this article, we demonstrate the utility of combining large, standardized data sets and comprehensive, standardized tests to evaluate the efficacy of different RNASeq normalization methods at different scales of analysis. We combined these tests into a single protocol to facilitate future research.

## Results

We performed two experiments to assess the validity of common normalization methods (see methods section) on a data set generated by the Sequencing Quality Control (SEQC) consortium. Experiment 1 was designed to quantitatively assess the relative contributions of biology and technology as sources of variability. Experiment 2 was designed to test the internal linear logic of each normalization method by analyzing individual genes from the same sequencing facility.

### Experiment 1: global assessment of normalization

**Fig. 1:**
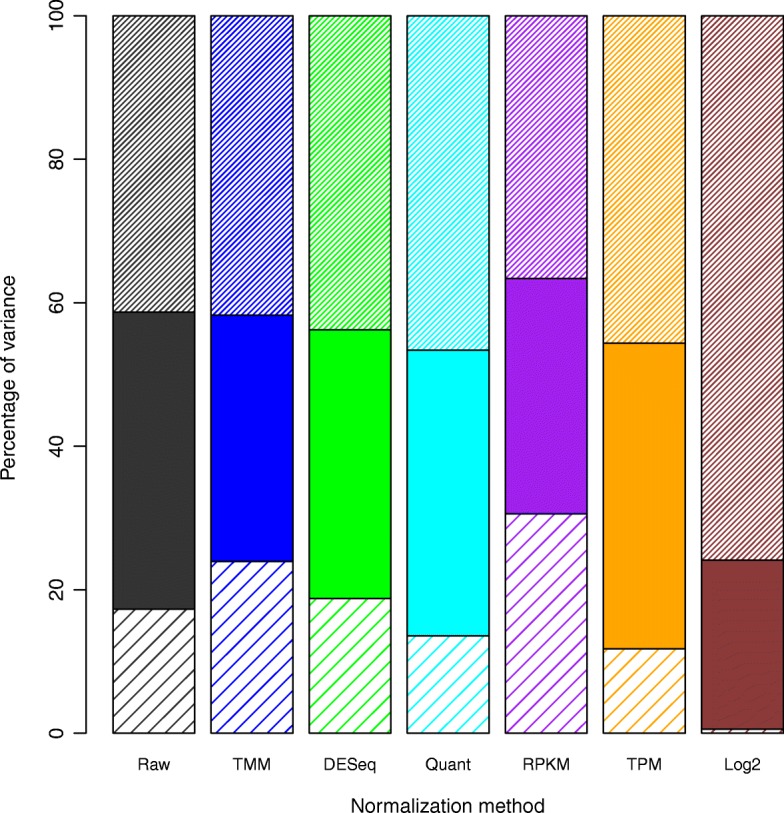
Bar Plot of Normalization Methods and their relative errors from a two-way ANOVA. The MSE for each of the features (site and biological condition) can be used to measure the amount of variance attributed to that specific feature. The top narrow striped bar is site dependent variability (batch effects); the solid bar is biological variability; and the bottom, wide striped bar is the residual variability

The three forms of variability that we identified are (1) site dependent batch affect, (2) biological differences, and (3) residual or unexplained. The decomposition into sources of variation as a proportion of total signal is presented in Table 1 and, as an overall stacked bar plot, in Fig. 1. We found that (94%) of the genes tested had significant association between site and gene expression and (37%) of genes tested had significant association between sample and gene expression using a two-way ANOVA.

**Table 1:** Percentage of total genes with a significant *p*-value after each normalization technique. Site *p*-values correspond to the association of site with gene expression and Sample p-value corresponds to the association of sample and gene expression.

	Raw	TMM	DESeq	Quant	RPKM	TPM	Log2
% site *p*-values<0.05	0.95	0.95	0.94	0.92	0.87	0.90	1.00
% sample *p*-values<0.05	0.37	0.27	0.35	0.34	0.36	0.49	0.69
Variance site *p*-values	1.40E-02	1.30E-02	1.39E-02	2.00E-02	2.73E-02	2.28E-02	4.44E-77
Variance sample *p*-values	8.73E-02	8.78E-02	8.61E-02	8.42E-02	8.62E-02	8.12E-02	6.37E-02
Median site *p*-value	2.28E-59	1.11E-64	5.22E-51	2.60E-58	1.64E-20	1.85E-35	0.00E+00
Median sample *p*-value	2.00E-01	2.98E-01	2.17E-01	2.30E-01	2.13E-01	5.71E-02	1.28E-05

Based on these results, TPM is the best performing normalization method because it increases the proportion of variation attributable to biology compared to the raw data (90% of genes had a significant association between site and gene expression and 49% of genes had a significant association between sample and gene expression). It is the only normalization method tested that meets this basic criterion increasing biological variability from 41% (raw) to 43% (TPM). TPM does increase site dependent error (Raw: 41% to TPM: 45%) but also reduces the residual variability (Raw: 17% to TPM:12%). This observation is important since residual variability is the worst form of the three types of error to have because it is created solely by uncontrollable experimental conditions. This is unlike biological variability, which is desired, or site dependent variability, which is traceable and therefore often correctable. Residual variability comes from non-desirable experimental problems that are not easy to identify or correct. TPM, Quantile, and Log2 are the only tested methods that reduced residual variability. However, based on our results, Log2 transformation reduces the biological signal to practically nothing making it one of the worst methods possible for normalizing data when the goal is downstream biological analysis. Quantile, though it reduces residual variability, also slightly reduces biological variability (Raw: 41%, Quantile: 40%).

If the majority of variability after normalization cannot be attributed to biology, then the majority of what the researcher is measuring is not grounded in underlying biological truth. Instead, they are measuring experimental bias either from batch effect or other non-biological sources of error. This is highly problematic if biological results are to be concluded from experiments where the majority of the measurable variability is not caused by biology. Consequently, a normalization method should increase the proportion of biological variability so that researchers are measuring biological truth and not experimental error when addressing their biological hypotheses.

### Experiment 2: effects of normalization on single genes

**Fig. 2:**
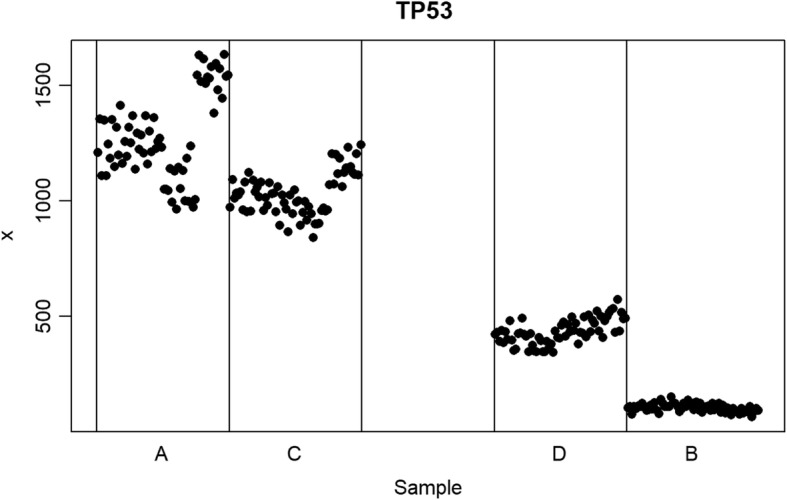
Raw read counts for the gene TP53 from the Australian Genome Research Facility site arranged by sample types (**a**, **c**, **d**, and **b**). The Y axis shows the read counts. The blank space in the middle represents where a 50–50 mixture of (**a** and **b**) would be located if one had been created and measured. By leaving this blank space, a visual interpretation can be made for the linearity between (**a** and **b**) by whether (**c** and **d**) mixture models fall on this linear line. If C or D do not fall on the linear relationship of A and B then the normalization method is imposing unwanted structure on the data. If all four samples (**a**, **b**, **c** and **d**) form a clear linear relationship then that normalization method is representing the true biological structure of the data

The second experiment was designed to test whether normalization preserved linearity by analyzing four individual genes chosen for their common study and/or use in medical sciences. TP53 (tumor suppressor), GAPDH (house-keeping gene), CD59 (hemophilia related), and POLR2A (RNA synthesis) were all used from the same site source (the Australian Genome Research Facility). We performed individual gene experiments by comparing two independent gene sample preps and their mixture models. This created a linear relationship between samples A and B, with mixture samples C (=75% A and 25%B) and D (=25% A and 75% B) lying on the linear fit (Fig. 2).

**Fig. 3:**
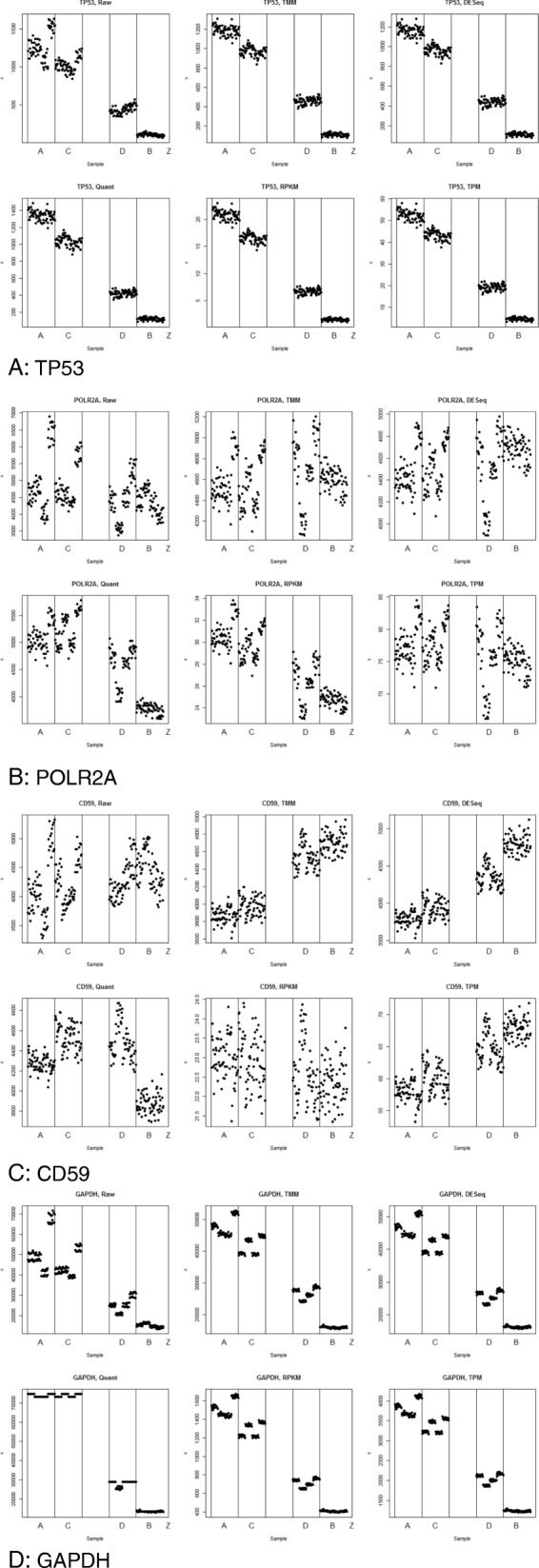
**a** TP53. **b** POLR2A. **c** CD59. d GAPDH

Results of experiment 2 are shown for four individual gene in Fig. 3a-d. These results illustrate several important patterns. First, no normalization method perfectly met the goal of normalizing data at the individual gene level at a single data source site. All normalization methods tested (see methods section) fell short of this goal; however, they fell short in different ways and to varying degrees. The quantile normalization repeatedly failed the linearity test, as demonstrated here by CD59 (Fig. 3c). This is a significant problem since it implies that these normalization methods imposed new structure on the data that should neither be there nor was originally present in the raw data. No normalization method should break the linearity rule under any circumstances given the mixture nature of the C and D samples and their inherent relationship to A and B.

Further, most methods did not address the batch effect problems created by the four library preps used within each sample (see methods section). As an example, comparing TMM and raw data it is readily observable that individual clusters representing different library preps are still evident after TMM normalization. This is a problem since normalization should remove such non-biologically founded artifacts in RNASeq experiments.

The gene GAPDH was not consistent between samples A and B. This indicates that GAPDH, often used as a control gene, is not an ideal gene for use as an internal control since its expression level varies widely between different tissue types as indicated in Fig. 3d. For this reason we recommend *not* using GAPDH either as a control gene for wet lab experiments or in any form of normalization procedures since its differential expression is likely to skew any normalization or control procedure.

Finally, of the biologically oriented normalization methods, transcripts per million (TPM) was amongst the highest performers. TPM did not introduce new or unwanted structure to the data. Further, it did reduce the noise generated from the library preps in all four of the gene cases. This is also true of the total read count normalization, a similar simple biologically oriented normalization technique. This leads to the observation that the most effective normalization method applied to RNASeq data should be the least complex method and should be biologically rather than purely mathematically or statistically driven.

## Discussion

In this paper we proposed a methodological protocol for the systematic evaluation of normalization methods for RNASeq analyses**.** We presented both a standardized data set designed to perform systematic evaluations along with two tests to determine the underlying validity of different normalization criteria. One of these criteria was the analysis of variance of site and sample type. Related to this we address another fundamental point that site dependent variability is quantifiable and therefore could be addressed during analysis especially when using joint distributions in linear models (like generalized linear models in both EdgeR and DESeq packages). These considerations should be taken into account when evaluating the effectiveness of normalization methods since theoretically a perfect model could completely eliminate the site dependent error downstream. In our case TPM had higher $\frac{site+ biology}{residual}$ and $\frac{biology}{residual}$ than all other methods except for Log2, which is just a simple transformation. However, since Log2 highly inflated site dependent error we conclude that TPM is the preferred method for normalizing RNASeq data.

While some methods tested better than others, the aim of this research was to offer a protocol for normalization evaluation; that is, other normalization methods not utilized in this article should be tested with this large standardized set of data and evaluated in this standard manner. To that end, we are publishing all of our code and data from this project in Supplemental files so that other researchers will be able to evaluate their own normalization methods for any RNASeq data. One limitation of our study was that it only applied to normalization methods for RNASeq data, which are platform specific. However, we have proposed a set of mathematical methods and tests that should translate to other platforms.

## Conclusion

Normalization is of vital importance to accurately interpret the results of genomic and transcriptomic experiments since normalization controls for experimental error while preserving biological truth. In this study we presented two tests to assess the validity of various normalization methods applied to a large-scale data set collected for systematic evaluation purposes. We tested different RNASeq normalization procedures and concluded that TPM was the most effective normalization method. More work, however, needs to be performed to optimize normalization methods for RNASeq data. The present effort helps pave the way for more systematic evaluations of normalization methods across different platforms. With our proposed protocol researchers can evaluate their own or other normalization methods that were not tested in this article to further improve the field of RNASeq normalization.

## Methods

### Data

The Sequencing Quality Control (SEQC) consortium is a large, diverse, and trusted collaboration that includes many participating academic, government, and industry partners. The organization is well established and has been evaluating transcriptomic technologies since 2006, when it was called the MAQC (microarray quality control) consortium [5]. The consortium’s current focus is the unbiased evaluation of RNASeq technologies from start to finish of the RNASeq pipeline and has even been used to study the effectiveness of spike-ins in normalization [6]. The consortium recently used well-characterized RNA samples to perform quality tests on various sequencers, microarrays, qPCR, genome annotations, and aligners regularly used in transcriptomics [5]. To attain results with the highest possible internal and external validity, the consortium mixed two samples (human reference RNA and human brain reference RNA) in known ratios (0:100, 25:75, 75:25, and 100:0) and used PCR validation to vet the various normalization methods being evaluated. Because of the complex nature of the study design, a large standardized set of runs was performed on multiple sequencing platforms across multiple sites “comprising >100 billion reads (10Tb), [providing] unique resources for evaluating RNASeq analysis for clinical and regulatory settings” [7]. From this data set, we used the read count files to conduct a comparative analysis of downstream normalization techniques regularly used specifically in RNASeq experiments.

The large, standardized data sets provided by SEQC facilitate the development of a systematic approach to evaluate the technologies used in RNA-Seq. Along with the aforementioned pipeline methods the data sets are well-suited for evaluating normalization methods because they were specifically designed for reproducible comparative analyses. Unlike past studies to evaluate normalization methods, ours is unique in the quality, consistency, completeness, and scope of the data, enabling us to perform a broad range of tests. The importance of using standardized methods, well-documented RNA, and extensive replicates cannot be overstated. Mixing samples in fixed ratios allows for an internal validation of the normalization techniques using linearity. Matching qPCR was conducted on all of the samples, facilitating evaluation against external “ground truth”. Cross-site read count files are also available, allowing for the evaluation of normalization across multiple sites.

The specific data used in our analysis was 10 data matrices consisting of raw RNASeq counts per transcript from the SEQC study, 10 random data matrices (uniformly randomized without replacement of the 10 SEQC data matrices), and one data matrix consisting of a random Poisson distribution of counts. Since all of the values contained within the 10 randomized files are the same, the overall distribution of the values is the same as the nonrandom files except that all of the patterns in the data have been randomized. In this way, the over-dispersion problem can be ignored for the 10 randomized datasets. Of the 14 original site files that SEQC generated, four had to be excluded from the analysis. Three of the files from the ROCHE 454 platform were excluded due to the lack of technical replicates (1 replicate per site per sample). Without technical replicates, the normalization methods, especially TMM and DESeq, will not function properly. Additionally, the New York Genomics (NYG) file had to be excluded from our analysis due to inconsistencies in annotation. Unlike the other Illumina and Life Sciences read count files, the NYG site used gene symbols instead of RefSeq IDs and included fewer genes. The following normalization methods were employed in Experiment 1 in addition to the raw count data:

### Experiment 1 Normalization methods: Between Site Variability Test

1. Raw counts (no normalization);
2. TMM - the trimmed mean of means, as implemented in edgeR [8];
3. The size factors, as implemented in DESeq [9];
4. RPKM, reads per kilobase transcript per million mapped reads [10];
5. TPM, transcripts per million mapped reads [11];
6. Quantile normalization [12];
7. Log2 transformation

These normalization methods were selected based on several criteria. They are most often used in other normalization method comparison studies and are the most commonly used in practice. They provide a variety of approaches to addressing the question of normalization. Two of the methods (Quantile and Log2) are purely mathematical non-biological approaches to normalization whereas TPM and RPKM are biological transformations based on transcript size. Specifically, Log2 may not be strictly defined as normalization but is included since it is so commonly used in research. The other two -- TMM and DESeq -- use a combination of both biologically based and mathematically based approaches to normalization. Although both methods are not designed to directly normalize data they are both commonly used methods for normalization and differential expression analysis, and thus were included in the study. Raw counts are used both as a control and because some researchers do not perform any normalization on the data prior to analysis. All of the normalization methods are implemented in R by our group with the exception of TMM (EdgeR package) and size factors (DESeq package).

### Experiment 1: Test of between site variability

This statistical test measures the amount of variability and identifies the source of that variability. There are three main sources of variability in genomic data: (1) site dependent or batch effect variability (2) biological variability, and (3) residual or other variability. Any normalization method should decrease the amount of variability attributable to site dependence and residual variability and thus increase the proportion of variability attributed to biology. To test this we performed a two-way ANOVA across our feature set where one variable was the different sites and the other was the four different samples, A, B, C and D. This allowed us to isolate the source of variability and its proportional weight of total variability.

After having performed the two-way ANOVA three different forms of error were identified; 1) site dependent error, 2) biologically dependent error and 3) residual error. Site dependent error is error from between different sites and institutions. Specifically these measurements were recorded as MSE (variance) and *p*-values for each gene across all samples. We took the median of both MSE p-value for inclusion in the figures and tables. Biological error is determined to be changes based on the biological differences of the four sample types. Residual error is any form of error that is not attributable either biology or site. Looking across the entire genome in increasing gene size we could access how each normalization method was able to deal with these three different forms of error. It is important to note that a good normalization technique should decrease site dependent and residual error while increasing the proportion of total signal that is attributable to biology. This is because biological signal is the only form of signal that we wish to preserve after normalization, so site and residual errors should, theoretically, decrease as a proportion of the total amount of error after normalization.

### Experiment 2 Normalization methods: Test of internal linearity

1. Raw counts (no normalization);
2. TMM - the trimmed mean of means, as implemented in edgeR [8];
3. The size factors, as implemented in DESeq [9];
4. RPKM, reads per kilobase transcript per million mapped reads [10];
5. TPM, transcripts per million mapped reads [11];
6. Quantile normalization [12];

We performed these individual gene experiments using only the Australian Genome Research Facility data to keep the site source consistent for this experiment. We used the same normalization methods as for use in experiment 1, with the exception of a log2 transform. This is because a log2 transform will not change any linear relationships in the data, and this experiment is designed to see how normalization affects internal linearity.

The four genes we selected were TP53, GAPDH, CD59, and POLR2A. TP53 was chosen since its relationship to cancer is well established. GAPDH was selected because it is commonly relied upon as a constitutively expressed housekeeping gene used as a control in many experiments. CD59 and POLR2A were chosen due to their high and consistent median expression across all four samples (A-D). For this reason the raw data does not indicate that there is any significant expression difference between A and B for either of these genes, making them good test cases.

## Data Availability

The datasets used and/or analyzed during the current study are available from the corresponding author on reasonable request.

## Abbreviations

CD59: Gene name for the MAC-inhibitory protein
DESeq: Differential expression analysis for sequence count data
GAPDH: Glyceraldehyde 3-phosphate dehydrogenase
MAQC: Microarray quality control
POLR2A: RNA Polymerase II Subunit A
RPKM: Reads per kilobase transcript per million mapped reads
SEQC: The Sequencing Quality Control
TMM: The trimmed mean of means
TP53: Tumor protein 53
TPM: Transcripts per million
